# From Trauma to Transformation: A Case Report of Interdisciplinary Rehabilitation of Fractured and Discoloured Maxillary Incisors

**DOI:** 10.7759/cureus.79233

**Published:** 2025-02-18

**Authors:** Yashica Dhingra, Ushmita Mehta, Ayushi Sharma, Mayur Kaushik, Sameer Ahmed, Ayush Kumar

**Affiliations:** 1 Periodontology and Implantology, Subharti Dental College and Hospital, Swami Vivekanand Subharti University, Meerut, IND; 2 Orthodontics and Dentofacial Orthopaedics, Subharti Dental College and Hospital, Swami Vivekanand Subharti University, Meerut, IND; 3 Conservative Dentistry and Endodontics, Subharti Dental College and Hospital, Swami Vivekanand Subharti University, Meerut, IND; 4 Prosthodontics and Crown and Bridge, Subharti Dental College and Hospital, Swami Vivekanand Subharti University, Meerut, IND

**Keywords:** electrosurgery, endodontics, orthodontics, prosthodontic rehabilitation, surgical template, trauma

## Abstract

Managing complicated crown-root fractures in the aesthetic zone presents significant clinical challenges, requiring a multidisciplinary approach to ensure both functional and aesthetic success. This case report describes the comprehensive rehabilitation of a 20-year-old male with discoloured and fractured maxillary incisors following trauma. The treatment plan involved four phases: endodontic therapy to address pulpal necrosis, orthodontic retraction to create adequate overjet, surgical template-guided crown lengthening using electrosurgery for precise tissue management, and prosthodontic rehabilitation with post-core-supported layered zirconia crowns. The collaborative efforts of endodontists, orthodontists, periodontists, and prosthodontists ensured meticulous planning and execution at each stage. Prefabricated glass fibre posts provided structural reinforcement, while the use of a surgical template facilitated controlled crown lengthening, maintaining biological width and achieving superior aesthetics. The integration of advanced materials and techniques resulted in a functionally stable, aesthetically pleasing outcome, highlighting the importance of interdisciplinary coordination in complex anterior dental restorations.

## Introduction

The restoration of endodontically treated teeth with complicated crown or crown-root fractures is a major challenge for dental practitioners because it requires a comprehensive and accurate diagnosis and treatment plan. Incorporating the expertise of various dental specialists ensures that all aspects of the patient's dental health are considered. In day-to-day life, we encounter complicated coronal fractures of permanent incisors, representing 11%-15% of all trauma to incisors, with 96% involving maxillary central incisors [1]. The management of such cases typically necessitates the placement of a definitive crown. Adequate overjet is essential, particularly in the esthetic region, to ensure optimal crown positioning. One method to achieve this is by retracting the lower anterior teeth. After orthodontic retraction, crown lengthening is performed, followed by the use of post and core-supported restorations [2].

One advanced technique employed for crown lengthening is electrosurgery, a method that uses high-frequency electrical currents to precisely remove soft tissue with minimal discomfort and efficient healing. Crown lengthening in the aesthetic zone requires meticulous planning and precise execution to achieve “pink-to-white aesthetics” [3]. Here comes the role of a collaborative approach between prosthodontists, who oversee the design and placement of the surgical guide/template by communicating detailed requirements and aesthetic goals, and periodontists, who perform the surgical aspects of the crown lengthening procedure [4]. Following these preparatory procedures, post-core crowns are typically used to reinforce the tooth structure and provide a stable base for the final crown, ensuring both durability and aesthetic harmony [5].

This case report explores the integrative approach of various dental specialists for managing root canal-treated teeth in the aesthetic zone, focusing on the sequential use of crown lengthening using a surgical guide, orthodontic retraction, and post-core crowns.

## Case presentation

**Figure 1:**
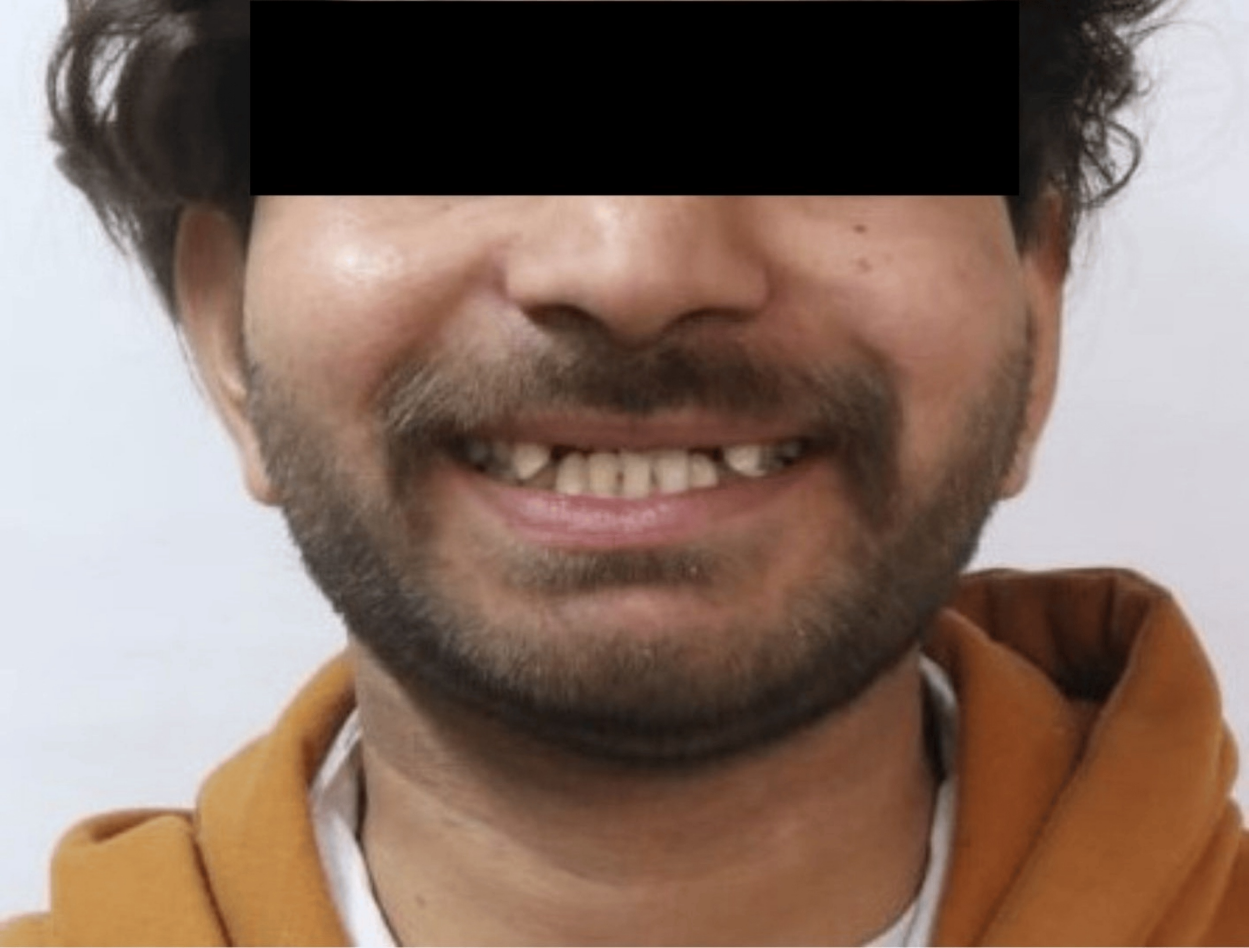
Pre-treatment photograph A 20-year-old male patient reported with a chief complaint of discoloured and fractured teeth in the upper front region of his jaw as a result of trauma around eight months ago due to a fall.

A 20-year-old male patient reported to the outpatient department of Conservative Dentistry and Endodontics with a chief complaint of discoloured and fractured teeth in the upper front region of his jaw as a result of trauma around eight months ago due to a fall (Figure 1). The patient exhibited no signs of swelling or pain. Clinical and radiographic investigations showed discoloured teeth with Ellis Class IV fractures involving teeth 11, 12, 21, and 22, affecting the pulp chamber without mobility and mild radiolucency around the periapical region. No response was observed with electric pulp stimulation and dry ice tests. Hence, the final diagnosis was "pulpal necrosis with asymptomatic apical periodontitis." After an in-depth discussion with the patient, it was planned to preserve the remaining tooth structure and proceed with an interdisciplinary treatment approach, organized into four phases.

Phase 1: Endodontic treatment

**Figure 2:**
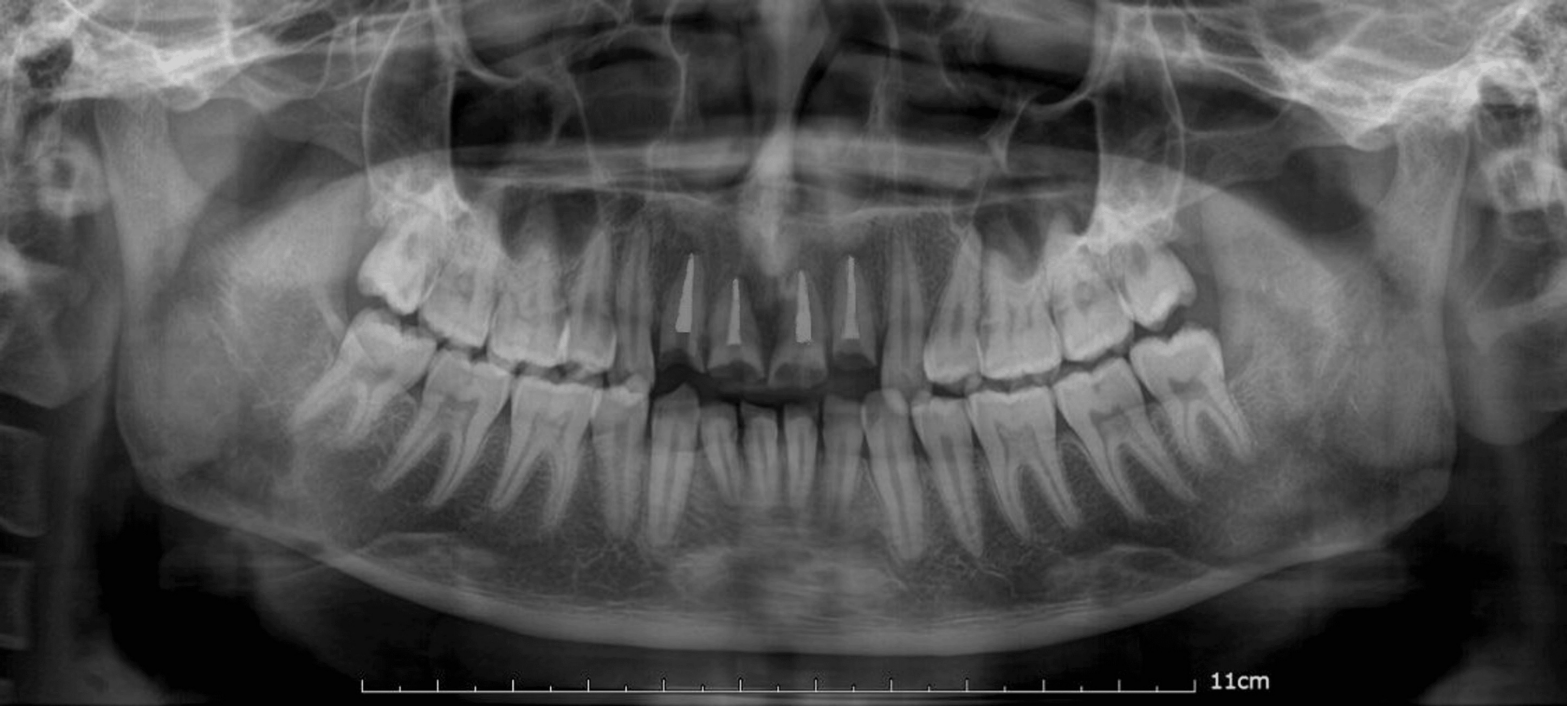
Post-treatment orthopantomogram Root canal treatment #12,11,21 and 22.

Access opening was carried out under rubber dam isolation. After establishing patency and the working length using an electronic apex locator and confirming radiographically, biomechanical preparation was performed using the step-back technique. The canals were irrigated with 5.25% sodium hypochlorite and 0.9% normal saline, with 2% chlorhexidine gluconate as the final irrigant. Calcium hydroxide was used as an intracanal medicament. Obturation was performed using lateral compaction of gutta-percha with CeraSeal Bioceramic sealer (Figure 2). A thorough full-mouth oral prophylaxis was performed before referring the patient to the orthodontic department.

Phase 2: Orthodontic retraction

**Figure 3:**
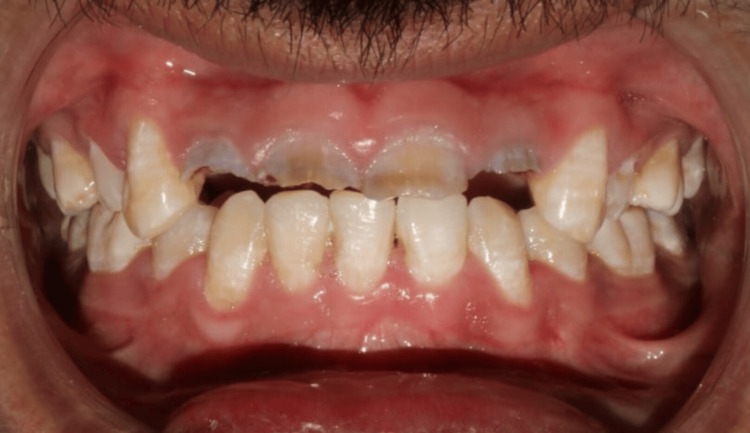
Orthodontic treatment Orthodontic retraction to achieve an overjet of 3.5 mm by extraction of #31 followed by closure of extraction space.

The patient had a history of prior orthodontic treatment, including the extraction of all first premolars. Intraoral examination revealed proclined lower incisors with reduced overjet. To address this, orthodontic retraction was planned, starting with the extraction of the left mandibular central incisor [6]. A 0.022" pre-adjusted edgewise appliance was used, followed by a 0.017" x 0.025" working arch wire with elastomeric chains to close the extraction space, achieving an overjet of 3.5 mm. The case was debonded after 12 months of treatment (Figure 3).

Fabrication of surgical template

**Figure 4:**
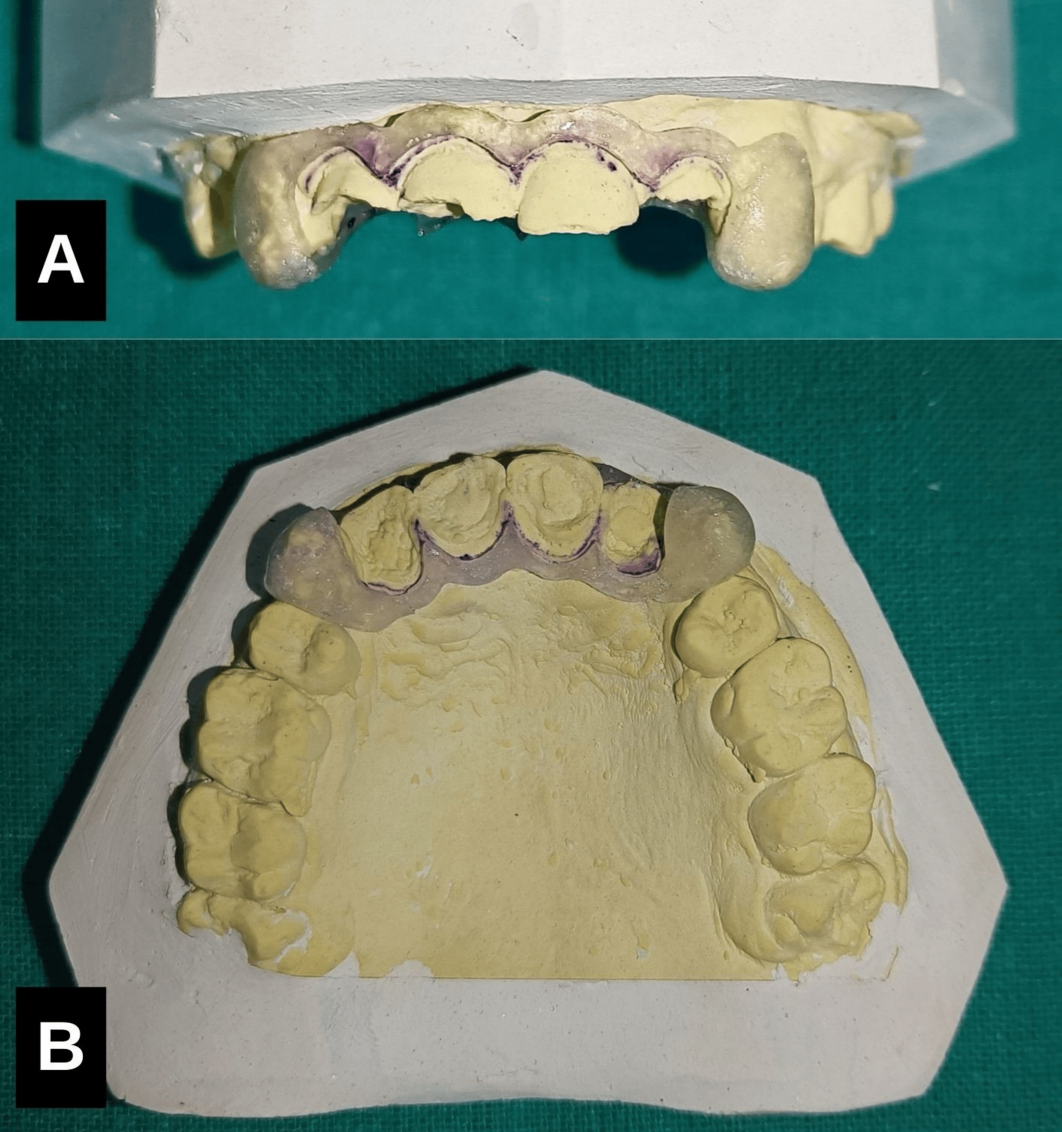
Fabrication of surgical template A surgical template was fabricated with heat-cured polymethyl methacrylate resin by marking the cementoenamel junction on the cast model. (A) Buccal aspect, (B) Palatal aspect.

A surgical template was fabricated by marking the cementoenamel junction on the cast model and planning gingivoplasty, 2 mm apical to the cervical margin. Wax was applied to create space for the template, and the template was constructed using heat-cured polymethyl methacrylate resin (Figure 4A, 4B).

Phase 3: Surgical template-guided periodontal crown lengthening

**Figure 5:**
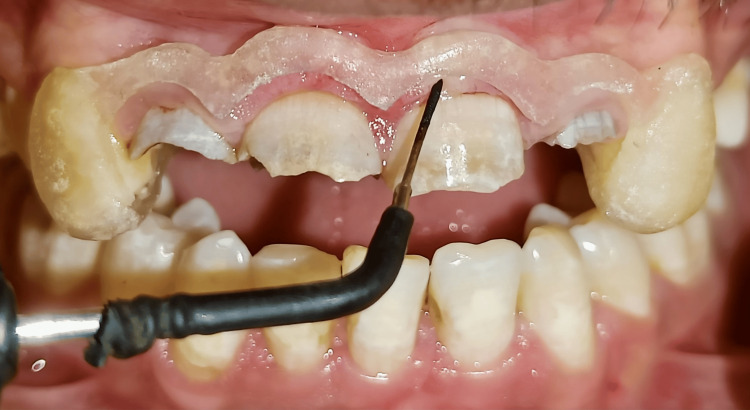
Surgical template Intraoral positioning of the surgical template for crown lengthening using electrosurgical unit.

**Figure 6:**
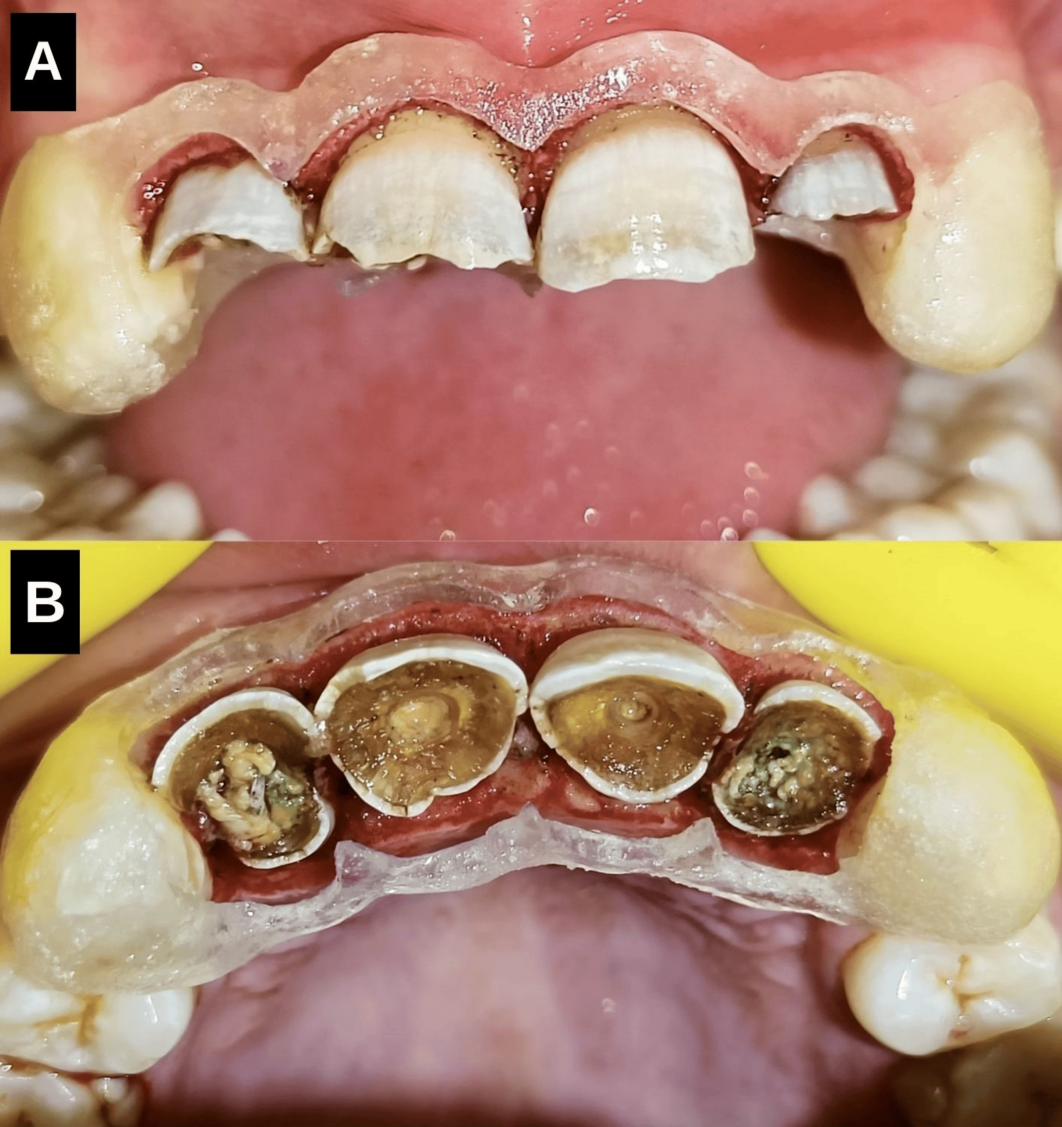
Surgical template-guided periodontal crown lengthening Surgical template was used to guide precise gingival tissue removal with an electrosurgical unit. (A) Buccal aspect, (B) Palatal aspect.

Under local anaesthesia, a surgical template was used to guide precise gingival tissue removal with an electrosurgical unit (Figure 5), ensuring minimal discomfort and efficient healing (Figure 6A, 6B). Post-operative oral hygiene instructions, antibiotics, and anti-inflammatory drugs were prescribed. Healing was satisfactory within 15 days with no complications.

Phase 4: Prosthodontic rehabilitation

**Figure 7:**
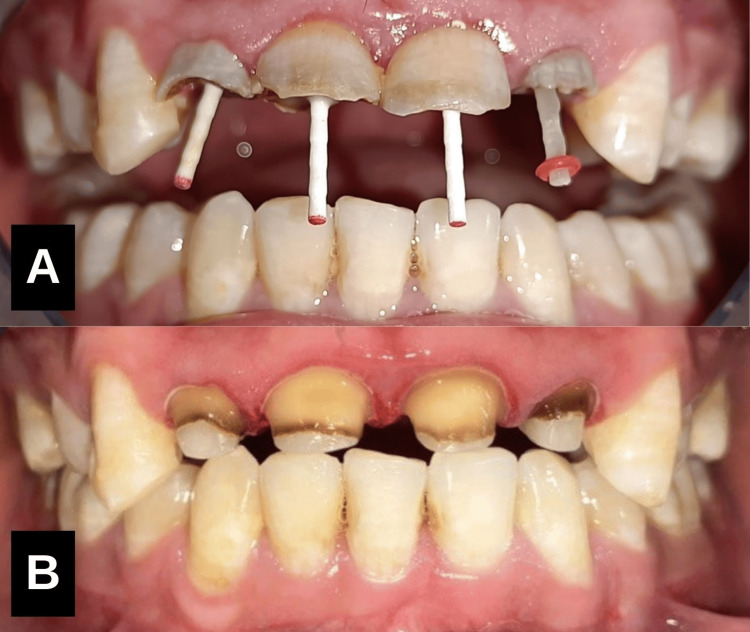
(A) Post and core, (B) Tooth preparation for all-ceramic full coverage crowns. (A) Post-space preparation was done and prefabricated glass fibre posts were placed, followed by core buildup with composite resin. (B) Tooth preparation was performed for layered zirconia full-coverage crowns.

**Figure 8:**
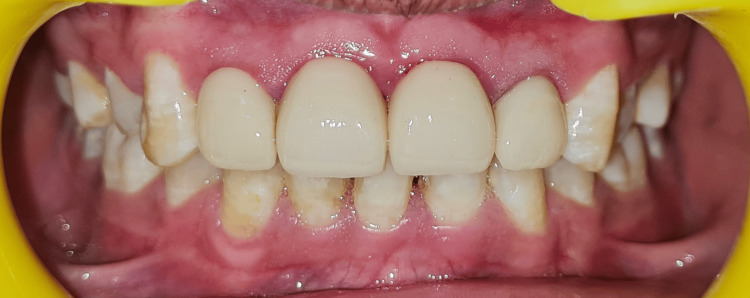
Full coverage crowns Cementation of layered zirconia crowns having superior aesthetics and strength, providing a natural appearance.

**Figure 9:**
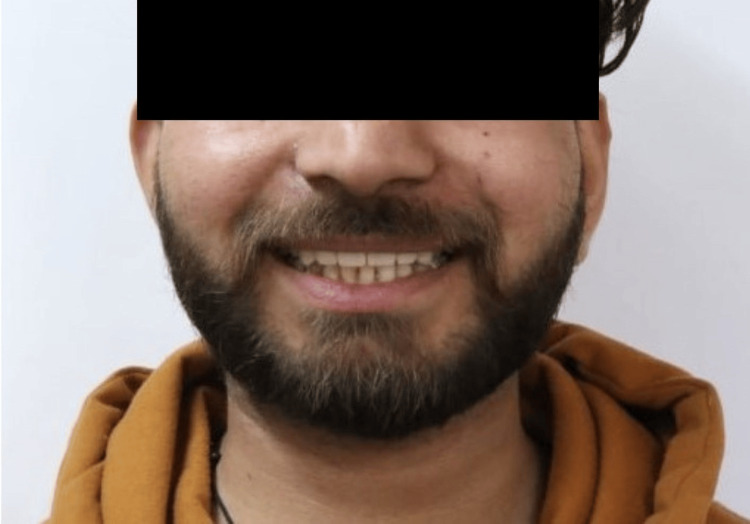
Post-treatment photograph The sequential integration of endodontic therapy, orthodontic retraction, periodontal crown lengthening, and prosthodontic rehabilitation restoring the patient's smile.

Three weeks post-crown lengthening, post-space preparation was done and prefabricated glass fibre posts were placed, followed by core buildup using composite resin (Figure 7A). Tooth preparation was performed meticulously for proper crown fit (Figure 7B). A facebow transfer was done on a semi-adjustable articulator and prosthesis was fabricated establishing a precise anterior guidance [7]. Layered zirconia crowns were chosen for their superior aesthetics and strength, providing a natural appearance (Figures 8, 9).

## Discussion

Crown-root fractures in the anterior region present significant clinical challenges due to their anatomical location and the need to balance functional restoration with aesthetic rehabilitation. These fractures often involve multiple dental tissues, including enamel, dentin, cementum, and pulp, leading to compromised structural integrity and functional impairments.

In this case report, the complexity is heightened by the proximity of the fracture line to the root within the aesthetic zone, making it imperative to maintain the biological width and ensure optimal periodontal health for long-term success. A multidisciplinary treatment approach was employed, incorporating the expertise of an endodontist, orthodontist, periodontist, and prosthodontist, each contributing to specific phases of the treatment plan [8].

Radiographic evaluation revealed that the fracture extended into the pulp, necessitating endodontic therapy. Advanced rotary file systems were utilized to enhance the efficiency of root canal debridement and to preserve the remaining tooth structure [9].

While literature commonly advocates orthodontic extrusion to elevate the fracture margin above the gingival level, this case involved orthodontic retraction to create adequate overjet, thereby improving accessibility for restorative procedures.

Preservation of periodontal health was a primary consideration to ensure the longevity of the restoration. A surgical template fabricated from polymethyl methacrylate (PMMA) was employed due to its biocompatibility, cost-effectiveness, ease of manipulation, reparability, and translucency. The combined use of electrosurgery with the surgical template facilitated precise tissue management, maintained the biological width, minimized surgical trauma, and contributed to superior aesthetic outcomes [10].

The extent of the fracture dictated the choice of final restorative materials. For extensive structural loss, a post-and-core system was indicated. Prefabricated glass fibre posts were selected for their ability to reinforce endodontically treated teeth, distribute occlusal forces evenly, and reduce the risk of root fractures. Their translucency and compatibility with resin-based luting agents also enhanced the aesthetic quality and bonding strength of the core buildup [11].

Layered zirconia crowns were chosen for their excellent mechanical properties and aesthetic potential. The inherent translucency and colour-matching capabilities of zirconia provided a natural appearance, making it an ideal choice for anterior restorations. The integration of a meticulously designed surgical template with high-quality zirconia crowns ensured a restoration that met both functional demands and aesthetic expectations [12].

Despite the advantages of using surgical templates and zirconia crowns, restoring crown-root fractures in the anterior region remains inherently complex. Precise diagnostic planning and meticulous execution are crucial, as any deviation during the surgical phase can adversely affect the final aesthetic outcome. This case underscores the importance of a comprehensive, interdisciplinary approach in managing such challenging dental scenarios.

## Conclusions

An interdisciplinary approach is essential for managing complicated crown-root fractures, especially in the aesthetic zone. The collaborative efforts of multiple dental specialists ensure comprehensive treatment, addressing both functional and aesthetic requirements. The sequential integration of endodontic therapy, orthodontic retraction, periodontal crown lengthening, and prosthodontic rehabilitation results in successful long-term outcomes, restoring the patient's smile and confidence.
